# Effects of two workload-matched high intensity interval training protocols on regulatory factors associated with mitochondrial biogenesis in the soleus muscle of diabetic rats

**DOI:** 10.3389/fphys.2022.927969

**Published:** 2022-09-23

**Authors:** Maryam Delfan, Alieh Vahed, David J. Bishop, Raheleh Amadeh Juybari, Ismail Laher, Ayoub Saeidi, Urs Granacher, Hassane Zouhal

**Affiliations:** ^1^ Department of Exercise Physiology, Faculty of Sport Sciences, Alzahra University, Tehran, Iran; ^2^ Institute for Sport and Health (iHeS), Victoria University, Melbourne, VIC, Australia; ^3^ Department of Anesthesiology, Pharmacology, and Therapeutics, Faculty of Medicine, University of British Columbia, Vancouver, BC, Canada; ^4^ Department of Physical Education and Sport Sciences, Faculty of Humanities and Social Sciences, University of Kurdistan, Sanandaj, Kurdistan, Iran; ^5^ Division of Training and Movement Sciences, University of Potsdam, Potsdam, Germany; ^6^ Movement, Sport, Health and Sciences Laboratory (M2S), UFR-STAPS, University of Rennes 2-ENS Cachan, Rennes Cedex, France; ^7^ Institut International des Sciences du Sport (2I2S), Irodouer, France

**Keywords:** diabetes mellitus, muscle metabolism, time-efficient exercise, mitochondrial adaptation, exercise training

## Abstract

**Aims:** High intensity interval training (HIIT) improves mitochondrial characteristics. This study compared the impact of two workload-matched high intensity interval training (HIIT) protocols with different work:recovery ratios on regulatory factors related to mitochondrial biogenesis in the soleus muscle of diabetic rats.

**Materials and methods:** Twenty-four Wistar rats were randomly divided into four equal-sized groups: non-diabetic control, diabetic control (DC), diabetic with long recovery exercise [4–5 × 2-min running at 80%–90% of the maximum speed reached with 2-min of recovery at 40% of the maximum speed reached (DHIIT1:1)], and diabetic with short recovery exercise (5–6 × 2-min running at 80%–90% of the maximum speed reached with 1-min of recovery at 30% of the maximum speed reached [DHIIT2:1]). Both HIIT protocols were completed five times/week for 4 weeks while maintaining equal running distances in each session.

**Results:** Gene and protein expressions of PGC-1α, p53, and citrate synthase of the muscles increased significantly following DHIIT1:1 and DHIIT2:1 compared to DC (*p* ˂ 0.05). Most parameters, except for PGC-1α protein (*p* = 0.597), were significantly higher in DHIIT2:1 than in DHIIT1:1 (*p* ˂ 0.05). Both DHIIT groups showed significant increases in maximum speed with larger increases in DHIIT2:1 compared with DHIIT1:1.

**Conclusion:** Our findings indicate that both HIIT protocols can potently up-regulate gene and protein expression of PGC-1α, p53, and CS. However, DHIIT2:1 has superior effects compared with DHIIT1:1 in improving mitochondrial adaptive responses in diabetic rats.

## 1 Introduction

Diabetes mellitus (DM) is a progressive metabolic disorder that is among the most common endocrine diseases in the world (Mellitus, 2005). Regardless of the type of diabetes, the primary pathogenesis of this chronic disease is blood glucose dysregulation which results in hyperglycemia, and is associated with insulin resistance or a lack of insulin secretion due to pancreatic β-cell dysfunction or cell death (Perez-Leighton et al., 2020; Rezaee et al., 2021). The prevalence of DM and its related complications continues to increase in most countries (Saeedi et al., 2020).

A multitude of secondary complications due to DM can arise in various organs, including the kidneys, brain, heart, and skeletal muscles (Forbes and Cooper, 2013). The inability of peripheral tissues, such as skeletal muscle, to utilize glucose is an important contributor to hyperglycemia (Abdul-Ghani and DeFronzo, 2010). Skeletal muscle is the primary organ for insulin-stimulated glucose uptake, as approximately 80% of blood glucose is absorbed by skeletal muscle (Deshmukh, 2016). Hence, any malfunction in the role of skeletal muscles in glucose homeostasis can lead to the onset and progression of diabetes.

Mitochondria are dynamic organelles in skeletal muscle and are involved in energy production and cellular homeostasis (Spinelli and Haigis, 2018). Molecular investigations into metabolic-related diseases indicate that mitochondrial defects are related to diabetes and associated consequences (Kelley et al., 2002; Petersen et al., 2004; Mogensen et al., 2007; Ritov et al., 2010), stressing the importance of gaining a deeper understanding of mitochondrial abnormalities in diabetes. Several transcriptional factors can regulate mitochondrial signaling pathways. Peroxisome proliferator-activated receptor gamma coactivator 1-alpha (PGC-1α), a “master transcriptional coactivator,” regulates mitochondrial biogenesis by interacting with other transcriptional factors (Fernandez-Marcos and Auwerx, 2011). A deficiency in PGC-1α is associated with abnormal glucose homeostasis (Leone et al., 2005; Handschin et al., 2007), making it a promising target for mitochondria-related metabolic disease therapy. The tumor suppressor protein p53 is another potential regulator of skeletal muscle mitochondrial function and biogenesis (Saleem et al., 2009). Decreased mitochondrial content and impaired respiratory function in p53 knockout mice underscores the importance of p53 in maintaining skeletal muscle mitochondrial health (Matoba et al., 2006; Park et al., 2009; Saleem et al., 2009). Additionally, the proper functioning of mitochondria is contingent on enzymatic activity. Citrate synthase (CS) is a mitochondrial enzyme whose inefficiency may be partly responsible for the pathogenesis of diabetes (Ørtenblad et al., 2005).

Unhealthy lifestyles, e.g., excess caloric intake and sedentary behavior, contribute to the onset and progression of diabetes, and current recommendations for the management of diabetes include lifestyle changes and performing regular exercise (Nelson et al., 2002). Metabolic adaptations to regular exercise depend on the specifics of the training programs employed (Nader and Esser, 2001; MacInnis et al., 2017; Granata et al., 2018a; Ghareghani et al., 2018). In this regard, exercise intensity is an important component of exercise prescription that influences skeletal muscle adaptation (MacInnis and Gibala, 2017; Granata et al., 2018b).

A lack of time for exercise often contributes to physical inactivity (Stutts, 2002). Thus, the emergence of time-efficient modes of exercise training, such as high intensity interval training (HIIT) featuring short near-maximal to maximal activity periods interspersed by shorter low-intensity activity periods or passive rest, is an alternative to traditional exercise training programs (Khakdan et al., 2020). Despite the reduction in total training duration, HIIT improves blood glucose, insulin sensitivity, and GLUT-4 expression in skeletal muscles (Little et al., 2011a; Karstoft et al., 2014).

Metabolic perturbations provide a potent stimulus for promoting mitochondrial biogenesis (Fiorenza et al., 2018), although it is unknown if the prescription of HIIT programs matched for volume and intensity but differing in recovery duration (i.e., 1 vs. 2 min) between intervals will have different effects on important modulators of mitochondrial biogenesis in diabetes. A HIIT protocol with a higher work-to-recovery ratio could be more metabolically challenging, leading to more pronounced changes in the intracellular environment (ADP/ATP turnover, reactive oxygen species (ROS), transient oxidative stress, Ca^2+^, and lactate accumulation), which in turn can lead to greater alterations of mitochondrial signaling pathways (Irrcher et al., 2009; Hoshino et al., 2016; Fiorenza et al., 2018). For example, increases in lactate stimulate mitochondrial biogenesis by upregulating PGC-1α (Hashimoto et al., 2007).

This study compared two volume-matched interval training protocols (i.e., 4 weeks; five sessions/week; 7.2 km running distance; 80%–90% of the maximum speed reached) performed at different work-to-recovery ratios on gene and protein expressions of p53, citrate synthase (CS), and PGC-1α in the soleus muscle of diabetic rats. We hypothesized that 1) both training protocols would improve key regulatory factors related to mitochondrial biogenesis compared to the untrained diabetic group, and 2) although both protocols were matched for volume and running distance, a HIIT protocol with a higher work-to-recovery ratio has greater effects on the regulators of mitochondrial biogenesis.

## 2 Material and methods

### 2.1 Experimental animals

Twenty-four male Wistar rats (8 weeks old, initial body mass 270 ± 20 g) were obtained from the Razi Institute, Iran. The animals were housed in a room with a temperature of 23–25°C, (45%–50% humidity and a light-dark cycle of 12–12 h). All animals were fed with a standard diet (Razi Institute, Karaj, Iran) *ad libitum*. Experiments followed the guidelines of the Animal Care Committee of the Tehran University of Medical Sciences (IR.SSRI.REC.1398.548). The animals were randomly divided into groups (6 rats per group) of a non-diabetic control (NC, injected with saline) and three other diabetic groups (injected with streptozotocin).

### 2.2 Induction of diabetes

Following acclimatization to the local environment and before beginning the training interventions, diabetes was induced in three groups of animals using the intraperitoneal injections of streptozotocin (60 mg/kg STZ, Sigma-Aldrich, St. Louis, MA, United States) dissolved in 0.05 M citrate buffer (pH 4.5), and nicotinamide (120 mg/kg NA, Sigma-Aldrich, St. Louis, MA, United States) dissolved in normal saline (Masiello et al., 1998; Szkudelski, 2012). This model has been used earlier to induce type 2 diabetes (Szkudelski, 2012). Levels of fasting blood glucose were measured 72 hours after the injection of STZ-NA in a drop of blood from a tail vein using a glucometer (Glucocard 01, Kyoto, Japan). A blood glucose concentration more than 200 mg/dl (11.1 mmol/L) was used to diagnose diabetes (Salwe et al., 2015). Treated rats were randomly assigned to either a diabetic control (DC), DHIIT1:1 that performed the HIIT1:1 program for 4 weeks, including 2 min of high intensity running and 2 min of recovery, or DHIIT2:1 that performed the HIIT2:1 program for 4 weeks, including 2 min of high intensity running and 1 min of recovery. The NC and DC groups did not perform any exercise training. They were only placed on a non-operative treadmill each day for 5 min.

### 2.3 Exercise protocols

The animals were first familiarized with running on a treadmill (Danesh Salar Iranian, Tehran, Iran) for 1 week, 10 min per day. They ran at a speed of 2 m/min on the first day and the speed was gradually increased on the following days. The maximum speed reached at the end of the week was calculated with a modified ramp test protocol, as previously described in detail (PITHON-CURI and TNC, 2007).

All rats in the training groups followed the HIIT programs for 4 weeks with five exercise days/week. The maximum speed was re-assessed on the sixth training day of every training week The maximum speed test was repeated at the end of the fourth week.

a) The HIIT1:1 program (2 min of high intensity treadmill running interspersed with 2 min of low-intensity recovery) started with 3 min warm-up running on the treadmill at 40% of the maximum speed reached, followed by the main training session. The intensity during the high intensity running gradually increased from 80% to 90% of the maximum speed reached over 4 weeks, and consisted of running for 2 min at 80%, 85%, and 90% of the maximum speed reached in the first, second, third, and fourth weeks, respectively. Each low-intensity period included 2 min of running at 40% of the maximum speed reached. After each training session, a cool-down period followed that included 3 min of running at 40% of the maximum speed reached.
b) The HIIT2:1 program (2 min of high intensity treadmill running interspersed with 1 minute of low-intensity recovery) consisted of a 3 min warm-up period that consisted of running at 30% of the maximum speed reached, followed by the main training session. Similar to the HIIT1:1 protocol (above), the HIIT2:1 protocol included 2 min of running at 80%, 85%, and 90% of the maximum speed reached in the first, second, third, and fourth weeks, respectively. Each low-intensity recovery period included running at 30% of the maximum speed reached for 1 min, followed by a cool-down period of 3 min of running at 30% of the maximum speed reached. Although the period of recovery differed between the two groups, the running distance in each session was the same in both DHIIT groups (total running distances of 7.2 km in both protocols). The exercise protocols are summarized in Table 1.

**TABLE 1:** The high intensity interval training protocols for both training groups.

Weeks of training	Group	Number of intervals per session	Intensity of each interval (% the maximum speed reached)	Total running distance (m)	Intensity of recovery (% the maximum speed reached)	Intensity of warm-up and cool-down (% the maximum speed reached)
1	DHIIT1:1	4	80	1,260	40	40
	DHIIT2:1	5	80	1,250	30	30
2	DHIIT1:1	4	85	1,440	40	40
	DHIIT2:1	5	85	1,435	30	30
3	DHIIT1:1	5	90	1,970	40	40
	DHIIT2:1	6	90	1,960	30	30
4	DHIIT1:1	5	90	2,620	40	40
	DHIIT2:1	6	90	2,620	30	30

### 2.4 Blood collection and tissue extraction

Fasting animals were anesthetized 48 h after the last training session with xylazine (10 mg/kg body weight, I.P.) and ketamine (90 mg/kg body weight, i.p.). Blood samples were obtained from the hearts and transferred into heparin-containing tubes. The serum was separated after centrifugation at 15°C at 3,000 rpm for 15 min and stored at −80°C for later analysis. Soleus muscles were harvested, immediately frozen in liquid nitrogen, and stored at −80°C for further experimentation.

### 2.5 Plasma biochemical analysis

The glucose oxidase method was used to calculate fasting blood sugar (FBS) using a quantitative glucose assay kit (Pars Azmoon, Karaj, Iran). An ultra-sensitive rat insulin ELISA kit (Mercodia, Uppsala, Sweden) was used to measure insulin levels.

### 2.6 mRNA expression by real-time PCR

Samples of soleus muscle tissue (50–60 mg) were homogenized in Trizol (Qiagen, Hilden, Germany) for analysis of mRNA expression levels. The quality and quantity of the extracted mRNA was evaluated using a nanodrop spectrophotometer (Thermo Scientific, Bremen, Germany). A transcriptor first-strand cDNA synthesis kit (Roche, Mannheim, Germany) was used to reverse transcribe total RNA for the synthesis of DNA using the manufacturer’s instructions. Gene expression levels were quantified using specific primers for PGC-1α, p53, and CS, and q polymerase chain reaction (qPCR) was carried out using the PCR master mix of 2X (Ampliqon, Odense, Denmark) with a final volume of 15 µl for each reaction in a real-time PCR system (Corbett, Rotor-Gene 6000, Qiagen, Hilden, Germany). All tests were run in duplicate. The target gene transcript levels were normalized relative to GAPDH that was used as a housekeeping gene. The PCR amplification occurred using the following conditions: 15 min for initial activation at 95°C, followed by 40 cycles of 15 s for denaturation at 95°C and 60 s for annealing/extension at 60°C (Table 2) (Kalaki-Jouybari et al., 2020).

**TABLE 2:** The primers sequence used in real-time PCR.

Gene name		Sequence of primer	Product size (bp)	Accession number
PGC-1α	Forward Reverse	CCG​AAG​AAC​CAT​CCG​ATT​GAA​G CCC​AAA​CCT​GAT​GGC​ATT​GTG	145	NM_031347.1
CS	Forward Reverse	GAG​ACT​ACA​TCT​GGA​ACA​C GAC​AGG​AAT​ATC​GTG​GAT​C	93	NM_016987.2
p53	Forward Reverse	CAAGAAGTCACAACACAT ATACTCAGCATACGGATT	127	NM_030989.3
GAPDH	Forward Reverse	TTC​TAG​AGA​CAG​CCG​CAT​C CAA​TGT​CCA​CTT​TGT​CAC​AAG​AG	139	NM_017008.4

### 2.7 Western blotting

Cellular proteins were extracted from the soleus muscle by homogenizing 70–100 mg of tissue in RIPA buffer (pH 7.4, 1% Triton X-100, 50 mM Tris–HCl, 0.2% SDS, 0.2% sodium deoxycholate, 1 mM Na-EDTA, and 1 mM PMSF) and treated with PMSF and protease inhibitor cocktail (Roche, Mannheim, Germany) to assess changes in protein abundance. The Bradford assay was used to determine total protein concentrations. After protein concentrations were determined, SDS–PAGE was used to separate equal amounts of protein from each sample, which were then transferred to PVDF membranes. The blocking process continued by incubation at room temperature for 2 hours with 5% non-fat dry milk or bovine serum albumin to unbind proteins sites in tris-buffered saline using 0.5% Tween-20. Primary antibodies against PGC-1α (Cell signaling, Beverly, MA, United States), p53 (SantaCruz, California, CA, United States), CS (Cell signaling, Beverly, MA, United States), and GAPDH (Cell signaling, Beverly, MA, United States) were used to incubate the blots overnight at 4°C. We used an enhanced chemiluminescent substrate to visualize target protein bands after incubation with second HRP-conjugated antibodies (horseradish peroxidase). Densitometry was performed with Image-J to analyze band densities (Ghareghani et al., 2018).

### 2.8 Statistical analysis

Data are presented as means and standard error of the mean (SEM) after normality and homogeneity of data were assessed using the Shapiro-Wilk and the Levene test. Differences between the non-diabetic and diabetic control groups were examined using independent *t*-tests. Training-induced effects were assessed with a one-way analysis of variance (ANOVA) computed at post, thus allowing us to determine whether significant differences between the groups were present at post. In case of a significant ANOVA output, the Tukey *post-hoc* test (least significant difference) for pair-wise comparisons was applied to identify paired groups that were statistically different. Values of *p* < 0.05 indicated statistically significant differences. The Statistical Package for Social Sciences (SPSS) 24 software was used to analyze the data.

## 3 Results

### 3.1 Effect of two different HIIT protocols on maximum speed reached

We measured changes in the maximum speed reached in response to training. The HIIT2:1 protocol enhanced the maximum speed reached by 64.4% (pre: 22.5 ± 2.0 m/min vs. post: 37.0 ± 1.5 m/min), while the HIIT1:1 protocol increased the maximum speed reached by 46.7% (pre: 22.5 ± 1.8 m/min vs. post: 33.0 ± 2.3 m/min). The effect of the HIIT2:1 protocol on maximal speed reached was greater than that of the HIIT1:1 protocol (*p* = 0.001).

### 3.2 Effects of the two different HIIT protocols on biomedical characteristics

Plasma biochemical parameters of animals after 4 weeks of training are shown in Table 3. Plasma levels of FBS were higher, and plasma insulin levels were lower in diabetic rats compared to non-diabetic control rats (*p* < 0.05). There was a difference between the diabetic groups for plasma levels of insulin and FBS (*p* < 0.05) after training for 4 weeks. Levels of FBS were lower in the DHIIT1:1 and DHIIT2:1 groups than in the DC group (*p* = 0.044 and *p* = 0.007, respectively), while FBS was similar in the DHIIT1:1 and DHIIT2:1 groups (*p* = 0.647). Only the DHIIT2:1 group showed a significant increase in plasma insulin levels compared to the DC group (*p* = 0.001), and there were no significant differences between the DHIIT1:1 and DC groups (*p* = 0.063). The difference was not significant between the DHIIT1:1 and DHIIT2:1 groups (*p* = 0.141).

**TABLE 3:** The plasma characteristics of the rats at the end of the fourth week.

Characteristics	Normal control	Diabetic control	DHIIT1:1	DHIIT2:1
FBS (mmol/L)	10.30 ± 0.70	31.51 ± 3.47^≠^	27.30 ± 1.85∗	25.87 ± 2.62∗
Insulin (ng/ml)	2.29 ± 0.14	0.38 ± 0.15^≠^	0.88 ± 0.11	1.28 ± 0.56∗

The data are presented as mean ± SD. ∗significant differences between diabetic control and DHIIT groups, ^≠^significant difference between normal control and diabetic control groups (*p* < 0.05). DHIIT1:1: Diabetic + HIIT1:1 program (2 min of high intensity treadmill running with 2 min of low-intensity recovery); DHIIT2:1: Diabetic + HIIT2:1 (2 min of high intensity treadmill running with 1 min of low-intensity recovery); FBS: Fasting Blood Sugar.

### 3.3 The effects of HIIT2:1 and HIIT1:1 protocols on regulatory factors related to mitochondrial biogenesis

#### 3.3.1 STZ-NA effects regulatory factors associated with mitochondrial biogenesis

**FIGURE 1:**
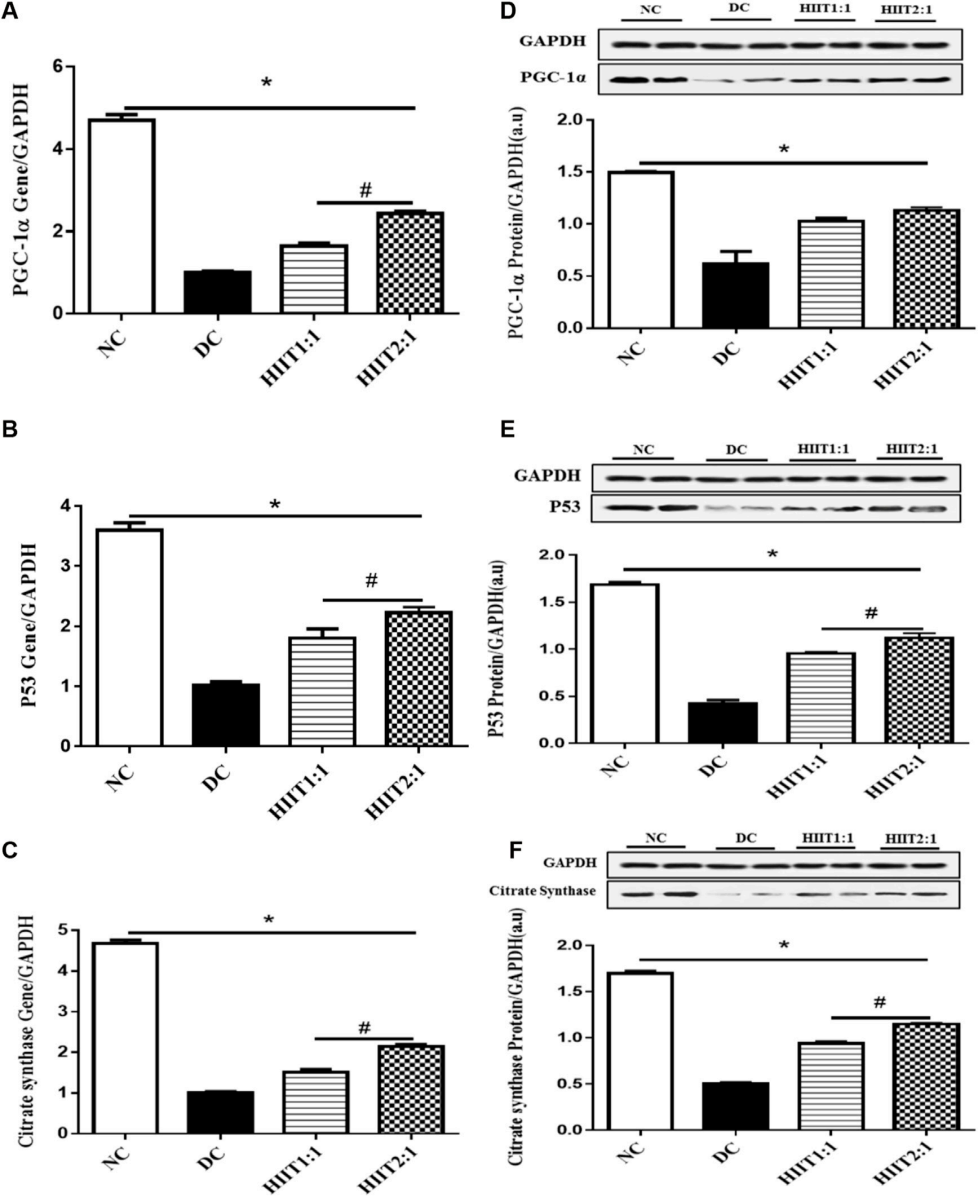
The effect of the HIIT1:1 and HIIT2:1 protocols on key regulatory factors related to mitochondrial biogenesis. **(A)** PGC-1α gene expression. **(B)** p53 gene expression. **(C)** Citrate synthase gene expression. **(D)** PGC-1α protein abundance. **(E)** p53 protein abundance. **(F)** Citrate synthase protein abundance. The data are presented as mean ± SEM. *: significant differences between control and other groups, #: significant differences between HIIT1:1 and HIIT2:1. HIIT: High Intensity Interval Training, *n* = 6 and rats trained for 4 weeks.

Control diabetic rats (DC) showed decreases in protein and gene expression levels of p53, CS, and PGC-1α compared to non-diabetic rats (*p*˂0.05) (Figure 1).

#### 3.3.2 PGC-1α

Levels of gene expression of PGC-1α in the soleus muscle were greater in both DHIIT1:1 and DHIIT2:1 rats compared to DC rats (*p* < 0.001) after 4 weeks of training. There were differences in the effects of DHIIT1:1 and DHIIT2:1 (*p* < 0.001), where a higher work-to-recovery ratio up-regulated PGC-1α mRNA expression (Figure 1A). Similarly, western blot analysis indicated that 4 weeks of exercise increased protein expression levels of PGC-1α in the DHIIT1:1 (*p* = 0.010) and DHIIT2:1 (*p* = 0.002) groups compared to the DC group. Protein expression levels of PGC-1α were similar in the DHIIT2:1 and DHIIT1:1 groups (*p* = 0.597) (Figure 1D).

#### 3.3.3 p53

Protein and gene expression levels of p53 in the soleus muscle were different between rats in the diabetic groups (*p* ˂ 0.05), with protein and gene expressions of p53 higher in the DHIIT2:1 and DHIIT1:1 groups compared to the DC group (*p <* 0.001). Furthermore, the content of both p53 mRNA (*p* = 0.046) and protein (*p* = 0.024) was greater after the HIIT2:1 protocol than after the HIIT1:1 protocol. These findings suggest that HIIT2:1 was more effective than HIIT1:1 in augmenting gene and protein expression levels of p53 (Figures 1B,E).

#### 3.3.4 CS

Protein and gene expression levels of CS were different between the DC and exercise-trained groups (*p* ˂ 0.05), with protein and gene expressions of CS greater following both HIIT protocols compared to DC (*p* < 0.001). The HIIT2:1 protocol augmented the gene and protein expression levels of CS more than did the HIIT1:1 protocol (*p* < 0.001) (Figures 1C,F).

## 4 Discussion

The main findings of our study are that: 1) Four weeks of both HIIT protocols with different work-to-recovery time ratios increased protein and gene expression levels of PGC-1α, p53, and CS in diabetic rats, but with the HIIT2:1 protocol being more effective than HIIT1:1, even though both exercise programs had the same volumes and total running distances (i.e., 4 weeks, five sessions/week; 7.2 km total running distance). 2) Both HIIT protocols reduced FBS, but only the HIIT2:1 protocol increased insulin levels in diabetic rats. 3) Both HIIT protocols increased maximal treadmill speeds reached, but the HIIT2:1 protocol was a more effective stimulus and contributed to the greater improvement in maximal treadmill speed. 4) Although there was a significant difference in the gene expression of PGC-1α between DHIIT groups, in contrast to our study hypothesis, protein expression levels of PGC-1α and plasma levels of FBS were similar in the DHIIT2:1 and DHIIT1:1 groups.

The therapeutic benefits of exercise training in patients with diabetes and metabolic syndrome is well-known (Colberg et al., 2010; American Diabetes Association, 2014; Gallo-Villegas et al., 2022). HIIT is a time-efficient alternative to moderate-intensity continuous training to improve various aspects of health in patients (Tjønna et al., 2008; Jung et al., 2015; Mendes et al., 2019) and in animal experiments (Chavanelle et al., 2017; Wang et al., 2017). We investigated the effects of two volume-matched HIIT protocols on mitochondrial biogenesis in the skeletal muscles of diabetic rats. The HIIT2:1 protocol stimulated gene and protein expression levels for both p53 and CS to a greater extent than the HIIT1:1 protocol. Despite significant differences in the PGC-1α gene expression levels between the two DHIIT groups, there were no differences in protein levels.

Low levels of PGC-1α expression is a potential pathogenic factor associated with diabetes (Mootha et al., 2003). Consistent with this, we observed lower protein and gene expression levels of PGC-1α in diabetic rats (Figures 1A,D). Mitochondrial adaptive responses are altered by different high intensity training programs (Gibala et al., 2009; Little et al., 2010; Little et al., 2011b). We provide novel findings on the effects of two different HIIT protocols on the regulation of PGC-1α protein and gene expression in diabetic rats. The observed increases in the expression levels of PGC-1α protein produced by HIIT were supported by other studies in obese rats (Khalafi et al., 2020), diabetic mice (Zheng et al., 2020), and humans (Hood et al., 2011). Although the molecular mechanisms by which HIIT induces mitochondrial signaling pathways are unclear, there is some evidence that the intermittent effort and the reoccurrence of metabolic fluctuations in the skeletal muscle may play a role (Combes et al., 2015). For instance, intermittent exercise (30 × 1-min intervals at 70% VO_2peak_ interspersed with 1-min of recovery periods) for 30 min further activated signaling pathways that regulate PGC-1α compared to a single bout of 30 min of continuous exercise performed at the same intensity (Combes et al., 2015). As a result, the number of transitions between work to recovery and the intermittent training pattern may be critical in inducing the mitochondrial biogenesis signaling cascade (Popov, 2020). Our findings suggest that one additional interval per session in the HIIT2:1, even in a volume-matched protocol, may have superior benefits in increasing gene expression of PGC-1α in the DHIIT2:1 group. However, we did not find a greater elevation in PGC-1α protein, and the higher increase in PGC-1α gene expression in the DHIIT2:1 group did not result in a higher increase in protein expression. The difference in protein expression would likely have been noticed if there had been additional weeks of training, especially given that other studies found an increase in PGC-1α protein in the muscle tissue of the animals following 8 weeks of HIIT (Zheng et al., 2020; Heiat et al., 2021). In response to frequent muscular contractions, fluctuations in the cytosolic concentration of numerous metabolites (including increased ADP:ATP ratios, Ca^2+^ flux, ROS generation, and redox status) regulate the expression of genes encoding proteins involved in mitochondrial biogenesis (MacInnis and Gibala, 2017). The metabolic sensors AMP-activated protein kinase (AMPK) and p38 mitogen-activated protein kinase (p38MAPK) phosphorylate PGC‐1α and regulate its transcriptional activity in skeletal muscle cells after high intensity training (Gibala et al., 2009; Bartlett et al., 2012). In addition to directly increasing PGC-1 activity *via* phosphorylation, AMPK indirectly increases PGC-1 activity in response to HIIT *via* SIRT-1, an NAD^+^-dependent deacetylase (Cantó et al., 2009; Gurd et al., 2010) in an intensity-dependent manner (Egan et al., 2010).

The tumor suppressor protein, p53, is a regulator of mitochondrial biogenesis, and its role as a guardian of the mitochondrial genome helps to maintain cellular homeostasis (Saleem et al., 2009; Itahana and Itahana, 2018). We report that the gene and protein expression levels of p53 were up-regulated by both HIIT protocols. In contrast, 8 weeks of aerobic training reduces p53 protein content in the skeletal muscles of Goto-Kakizaki rats that develop early insulin resistance and type 2 diabetes (Qi et al., 2011). Potential reasons for these conflicting results may be differences in the induction of diabetes, the sub-strain of animals, and training protocols. Because of the multifunctional role of p53, its expression depends on the cellular environment and the duration of the imposed stress (Horn and Vousden, 2007; Vousden and Prives, 2009). Mitochondrial transcription factor A (Tfam) is involved in integrating, repairing, and regulating mtDNA transcription. Contractile activity of skeletal muscles causes p53 to translocate to the mitochondria, where it binds with Tfam to enhance mtDNA transcription favorably (Saleem and Hood, 2013), while activating p38MAPK and AMPK after exercise phosphorylates p53 at Ser15, resulting in its stability and activation (She et al., 2000; Jones et al., 2005). PGC-1α contains a proposed p53 binding site in its promoter region, where binding e.g., after exercise, stimulates its transcription (Irrcher et al., 2008). Mitochondrial adaptive responses can be influenced by the nature of the exercise program (i.e., intensity, volume, and duration), with a role for training intensity in the up-regulation of regulatory factors associated with mitochondrial biogenesis (Egan et al., 2010; Granata et al., 2016; Granata et al., 2017). Our exercise program HIIT2:1 consisted of 5–6 × 2-min bouts at 80%–90% of the maximum speed reached during an incremental treadmill test, interspersed with 1-min active recovery periods at 30% of the maximum speed from the same test. Thus, even though rats in both groups ran equal distances, the rats in the DHIIT2:1 group ran ∼70% of the total training time at a high intensity and ∼30% of the training time at a low-intensity during each session. Rats in the DHIIT1:1 group ran ∼56% of the total training time at a high intensity and ∼44% of the training time at a low-intensity during each exercise session. Therefore, HIIT2:1 appears to be a stronger exercise stimulus for mitochondrial biogenesis because it underpins greater mitochondrial gene expression in the DHIIT2:1 group.

CS is a key mitochondrial enzyme involved in energy-producing metabolic pathways, and decreased CS activity is associated with insulin resistance and impaired lipid metabolism in skeletal muscles (Ørtenblad et al., 2005; Alhindi et al., 2019). Our study indicates that HIIT increased protein and gene expression levels of CS compared to untrained diabetic rats are supported by other findings that show decreases in CS protein levels in diabetic rats are reversed by 4 weeks of exercise training (Irrcher et al., 2008), and that CS mRNA levels are increased by interval walking training in individuals with type 2 diabetes (Karstoft et al., 2014). Given the increase in TCA cycle metabolites/intermediates flux in skeletal muscle during exercise (Gibala et al., 1998), it appears that intense bursts of exercise with fluctuations in ATP turnover increases levels of oxidative enzymes such as CS. Our study comparing two HIIT protocols indicated that the HIIT2:1 program caused greater increases in the protein and gene expression levels of CS than the HIIT1:1 program. Oxygen supply to cells and the relative demands on specific metabolic pathways in the muscle are affected by manipulating the duration of work-to-rest periods. It has been suggested that work intervals following a more extended recovery period begin with a lower metabolic rate (Schoenmakers and Reed, 2019). Thus, greater metabolic stress when exercising with a shorter recovery duration may provide a more effective exercise stimulus and appears to contribute to a greater improvement in this mitochondrial enzyme gene expression in the DHIIT2:1 group compared to the DHIIT1:1 group (Fiorenza et al., 2018).

Our results indicate that both DHIIT training protocols reduced FBS levels to a similar extent, suggesting that either a 1 or 2 min of recovery time improved fasting blood glucose levels and reduced FBS levels in both groups to the same extent. These observations are consistent with previous findings that HIIT reduces hyperglycemia in diabetic patients (Little et al., 2011a; Alvarez et al., 2016) and rodents (Chavanelle et al., 2017; Zheng et al., 2020). Insulin-stimulated GLUT-4 translocation is disrupted in diabetes, and exercise training stimulates the translocation of GLUT-4 to the muscle cell membrane in diabetic patients (Kennedy et al., 1999; O'Gorman et al., 2006). In addition to boosting insulin secretion and insulin-stimulated glucose uptake, HIIT also reverses hyperglycemia in an insulin-independent manner, involving contraction-mediated glucose uptake, increased GLUT-4 content, and improved muscle blood flow, all of which increase glucose delivery to active muscle (Little et al., 2010; Chavanelle et al., 2017; Dela et al., 2019). Another explanation for the efficacy of high intensity training is that muscle glycogen depletion following HIIT and subsequent glycogen resynthesis improves insulin sensitivity (Metcalfe et al., 2012). The findings of Madsen et al. (2015) that 8 weeks of HIIT did not change insulin secretion in patients with T2D is consistent with our study where the DHIIT1:1 group did not change insulin levels (although there were improvements in FBS), suggesting that the HIIT1:1 protocol may reduce FBS in an independent-insulin manner (Cartee, 2015; Sylow et al., 2017).

The maximum treadmill speed reached was improved by both the HIIT2:1 and HIIT1:1 protocols, although greater improvements occurred with the HIIT2:1 protocol. Exercise stimulates skeletal muscle mitochondrial biogenesis pathways and improves mitochondrial function (de Andrade Soares et al., 2020). Other studies reported that PGC-1α transgenic mice reached a higher maximum speed during exercise (Calvo et al., 2008), and that PGC-1α-b-mediated increases in mitochondrial biogenesis in skeletal muscles enhanced exercise capacity and peak oxygen uptake (Tadaishi et al., 2011). Additionally, increases in muscle oxidative enzymes during high intensity training decreased the time required to complete a set amount of work (Gibala et al., 2006). An increase in energy demand in response to intensive contractile activity may result in more mitochondrial adaptation to effectively address the increased energy requirements (Finck and Kelly, 2006), contributing to better performance and maximal treadmill speed. Although there was no difference in PGC-1α protein expression between the two exercise groups, the greater improvement in maximum speed in DHIIT2:1 can most likely be attributed to the higher protein expression of CS as a key enzyme responsible for oxidative metabolism and p53 as a regulatory protein involved in mitochondrial homeostasis. It is possible that both HIIT protocols, particularly the greater protein expression of CS and p53 with HIIT2:1, enhanced performance by elevating energy production capacity and increasing ATP availability for muscle contractions (Grassi et al., 2015). Furthermore, an increase in workload intensity corresponds to increased motor unit recruitment (Dudley et al., 1982). It is likely that HIIT2:1 leads to a greater improvement in maximal treadmill speed, partly by affecting the metabolic profile of muscle fibers.

## 5 Limitations

Our study has some limitations, including the lack of additional measures of mitochondrial adaptations (e.g., CS activity, mitochondrial respiratory function, etc.,) after various interventions. Furthermore, mitochondrial copy number, and tissue cross-sections were not measured. Future studies could evaluate the effects of two HIIT protocols on factors such as CS activity, mitochondrial respiratory function, and mitochondrial content.

## 6 Conclusion

The results of our study highlight skeletal muscle adaptations to different exercise stimuli in diabetic rats. Moreover, an increase in the work-to-rest ratio during HIIT appears to have a greater impact on mitochondrial adaptations in skeletal muscle. Therefore, the manipulation of the work:rest ratio may result in improved mitochondrial adaptations in diabetic patients. Future studies should investigate whether these findings can be translated to human beings as well.

## Data Availability

The original contributions presented in the study are included in the article/Supplementary Materials, further inquiries can be directed to the corresponding authors.
